# A Novel Serum Inflammation Risk-Index (SIRI-RT)-Driven Nomogram for Predicting Secondary Malignancy Outcomes Post-Radiotherapy

**DOI:** 10.3390/cancers17081290

**Published:** 2025-04-11

**Authors:** Sonia Gandhi, Sudhir Chandna, Vijayakumar Chinnadurai, Pankaj Vidyarthi

**Affiliations:** Institute of Nuclear Medicine and Allied Sciences, Delhi 110054, India; sss.gandhi@gmail.com (S.G.); vijayakumar.hqr@gov.in (V.C.);

**Keywords:** MIMIC-IV database, prognosis, nomogram, radiation-induced secondary malignancy

## Abstract

Radiation is often used as the primary treatment for a range of cancers. Nonetheless, its ability to trigger secondary tumors has emerged as a significant issue. Therefore, gaining insight into and predicting radiation-induced secondary cancers is essential for enhancing the long-term prognosis of cancer survivors. Background and Objectives: Previous studies have identified several factors; however, research on the use of serum-based inflammatory markers as prognostic tools for predicting radiation-induced secondary malignancies is limited. Investigating the potential of serum-based inflammation prognostic scores could provide a minimally invasive and affordable method for the early prediction of secondary malignancies. Methods: We retrospectively analyzed a patient cohort with radiation-induced secondary malignancy from the electronic database MIMIC-IV to investigate whether a serum-based inflammatory marker score can serve as a predictive tool. Results: This study seeks not only to assess the efficacy of the risk score, but also to develop a clinical utility tool nomogram for predicting the occurrence of radiation-induced secondary cancers. A RISM of 4.28% was observed in a cohort from the MIMIC-IV database using SIRI-RT as a risk index, with the Charlson comorbidity index, chemotherapy, and creatinine levels as significant confounding risk factors. Conclusions: Our study suggests that elevated serum-based inflammation prognostic scores and the nomogram developed herein can be used to predict a greater likelihood of developing secondary malignancies following radiation therapy.

## Simple Summary

Radiation is a key treatment modality for various cancers, and understanding its potential to cause secondary malignancies is crucial for improving the long-term outcomes of cancer survivors. Systemic inflammatory mechanisms elicited by radiotherapy may be important prognostic markers for predicting radiation-induced secondary malignancies (RISMs). In this study, we develop a novel prognostic scoring system (SIRI-RT), followed by nomogram construction, to predict the risk of developing post-irradiation carcinogenesis based on inflammation-related serum parameters recorded in the retrospective multi-center cohort of the MIMIC-IV database. This study identifies a 4.28% incidence of RISMs in a group from the MIMIC-IV database, utilizing SIRI-RT along with the Charlson comorbidity index, with chemotherapy and creatinine levels serving as significant confounding risk factors. Notably, chemotherapy and creatinine appear to be more protective than harmful. Implementing these scoring systems in clinical settings could offer an economical, non-invasive, and dependable clinical approach for predicting the progression free survival post-irradiation.

## 1. Introduction

According to the GLOBOCAN database of International Agency for Research on Cancer (IARC), there were approximately 19.3 million new cancer cases and 10.0 million cancer-related deaths globally in 2020 [1]. This classification has prompted calls for decreased exposure to radiation and enhanced education regarding its health hazards. Multiple meta-analyses have confirmed that radiation therapy, while effective in treating cancer, can trigger inflammation and elevate the risk of secondary cancers. In breast cancer patients receiving whole breast irradiation, pneumonitis is affected by systemic inflammation-immune status (SIS). Individuals with elevated post-radiation SIS scores demonstrated increased susceptibility to radiation-induced pneumonitis. The incidence rate in this group was 72.7%, compared to 54.5% in those with lower scores [2]. Moreover, chronic inflammation, a known seventh hallmark of cancer development, substantiated by epidemiological research, demonstrated positive correlations between inflammatory markers, such as CRP, and various cancers, including breast, colorectal, and lung cancers. Patients undergoing cranial radiation experience a 7–10-fold higher risk of developing secondary CNS tumors, with a 20-year cumulative incidence ranging from 1.03% to 28.9%. The latency period for these tumors may vary from 5 to 10 years in gliomas to approximately 15 years after irradiation [3]. Similarly, solid tumors outside the CNS generally exhibit latency periods of 5–15 years post-irradiation [4,5].

Exposure to radiation initiates a series of cellular and molecular processes that go beyond direct DNA damage. A key pathway involved in the development of radiation-induced cancer is persistent inflammation. This ongoing inflammatory response after radiation exposure has been found to foster a microenvironment conducive to tumor growth by encouraging genomic instability, enhancing the formation of new blood vessels, and aiding the evasion of the immune system, all of which play significant roles in the onset, progression, and metastasis. [6,7]. Ionizing radiation specifically triggers the NF-κB signaling pathway, which plays a crucial role in regulating inflammatory and immune responses [4,5]. Once activated, NF-κB enhances the production of inflammatory cytokines such as IL-1β, IL-6, IL-8, and TNF-α, thereby promoting a prolonged pro-inflammatory condition that can result in chronic inflammation and potentially lead to malignant transformation [6,8].

Furthermore, chronic inflammation caused by radiation results in the generation of reactive oxygen species (ROS), which exacerbates DNA damage, leads to genomic instability, and activates oncogenic pathways [9]. For example, increased C-reactive protein (CRP) levels are known to indicate systemic inflammation and are associated with a higher risk of cancer, greater tumor aggressiveness, and unfavorable prognosis in various cancers, including those secondary to radiation exposure [10].

Neutrophils and lymphocytes have dual functions in both cancer development and anti-tumor immunity. A higher neutrophil-to-lymphocyte ratio (NLR), which signals systemic inflammation, is consistently linked to poor cancer outcomes, highlighting the disparity between pro-inflammatory actions (neutrophils) and anti-tumor immune responses (lymphocytes). This imbalance can promote tumor advancement by aiding immune evasion and tumor growth, particularly in patients undergoing radiation therapy who remain in a state of chronic inflammation [11,12]. Platelet activation also plays a role in cancer metastasis by shielding circulating tumor cells from immune system attacks and aiding the establishment of metastatic niches. Elevated platelet levels and substances originating from platelets have been linked to a higher risk of metastasis and poorer cancer survival outcomes, highlighting the importance of platelet-based markers in secondary malignancies caused by radiation [13].

As discussed above, inflammation plays a crucial role in tumor initiation, progression, and staging [14]. Within the tumor microenvironment, it facilitates malignant cell growth and survival, promotes blood vessel formation, aids metastasis, impairs adaptive immunity, and reduces the effectiveness of chemotherapy drugs [15]. For patients exposed to radiation, an increase in both the presence of local immune cells and systemic inflammatory responses may serve as significant indicators of disease progression and outcome [16]. Systemic inflammation has consistently been associated with poor survival outcomes in patients with metastatic cancer across multiple studies. Biomarkers found in the peripheral blood before treatment offer insights into a patient’s initial state of inflammation and immunity. These indicators are considered potential prognostic factors because of their accessibility in clinical settings [17]. Ratios derived from various biochemical or blood markers commonly measured in standard blood tests can be used to assess systemic inflammation [18]. Some of these variables include C-reactive protein (CRP) [19], ratio of neutrophils to lymphocytes (NLR) [20], geriatric nutritional risk index (GNRI) [21], and advanced lung cancer inflammation index (ALI) [22]. Researchers have attempted to develop new scoring systems by integrating established prognostic indicators to forecast outcomes and inform clinical decision-making.

In patients with incurable colorectal liver metastases, the modified Glasgow Prognostic Score (mGPS), derived from C-reactive protein and albumin levels, was found to be an independent predictor of poor survival [23]. Similarly, elevated C-reactive protein levels independently predicted worse survival for individuals with both cancer-specific and relapse-free renal cancer [24]. Other inflammatory markers, such as neutrophil-to-lymphocyte ratio (NLR), platelet-to-lymphocyte ratio (PLR), and novel indices like the Systemic Immune-Inflammation Index (SII), have also been associated with poor prognosis in various cancers. In patients with colorectal cancer, a high SII is linked to poorer overall survival [25]. For prostate cancer, elevated NLR and Systemic Inflammation Response Index (SIRI) were associated with increased prostate-cancer-specific mortality, particularly in African American men [26]. Some studies have found contradictory results. In patients with esophageal cancer, myosteatosis (increased muscle fat) was associated with improved progression-free and overall survival, while systemic inflammation (NLR > 2.8) predicted worse outcomes [27]. This highlights the complex interplay between body composition and the inflammatory status in cancer prognosis.

Extensive evidence supports the biological plausibility of systemic inflammation as the mechanistic driver of radiation-induced carcinogenesis. Multiple studies across different cancer types have consistently demonstrated that markers of systemic inflammation are associated with poor survival outcomes in metastatic cancer. These results indicate that evaluating systemic inflammation could be beneficial for categorizing risk and determining treatment strategies in patients with metastatic cancer [28,29]. However, the available scoring systems using clinical markers are not applicable to radiation-exposed patients with secondary malignancies.

This study aims to develop a new prognostic scoring system that predicts the risk of developing radiation-induced secondary malignancies, based on inflammation-related serum parameters recorded in a retrospective multi-center cohort. Implementing these scoring systems in clinical settings could offer an economical, non-invasive, and dependable approach to detect secondary malignancies in their early stages, before resorting to specific biomarkers. This approach would not only validate disease advancement, but also facilitate prompt well-informed clinical choices to improve disease-free survival through timely intervention.

## 2. Materials and Methods

### 2.1. Data Source

Data on inflammation markers in the serum of patients with radiation-induced secondary cancer were sourced from the MIMIC-IV database (version 2.2). This database includes records of over 65,000 ICU admissions and more than 200,000 emergency department visits, totaling approximately 425,087 patient admissions from 2008 to 2019. MIMIC-IV is a product of collaboration between the Beth Israel Deaconess Medical Center (BIDMC) and the Massachusetts Institute of Technology (MIT). The data, collected at BIDMC during routine clinical care, are de-identified, processed, and made available to researchers who have completed human research training and signed a data use agreement. The BIDMC’s Institutional Review Board approved the sharing of these research resources and waived the need for informed consent. The creation of MIMIC generally involves three steps: data acquisition, transformation, and de-identification. Informed consent was not required as the patient data in this database are anonymized. Available information includes measurements, orders, diagnoses, procedures, treatments, and de-identified free-text clinical notes. 

We participated in courses from the US National Institutes of Health (NIH) and were authorized to access the database (Ref No.: R01EB030362).

### 2.2. Study Population and Data Acquisition

The International Classification of Diseases, ninth edition (ICD-9) and tenth edition (ICD-10), code 2015, were used to screen the database for patients with secondary malignant neoplasms following radiation therapy. Data were extracted using the Structured Query Language (SQL) program in Big Query on the Google Cloud Platform.

### 2.3. Variable Selection and Data Preprocessing

Using hadm_id, chartdate, admittime, icd_code, icd_version, and long_title, the data were extracted from the MIMIC-IV database. To ensure that the cohort analyzed for secondary malignancy was post-radiotherapy, the values corresponding to the variables considered in the analysis were obtained from the patient charts documented after radiation exposure using the field chart time from the database. Age, gender, comorbidities, vital signs, laboratory parameters, severity score system, and survival data were used as the clinical and demographic variables. Comorbidities including hypertension, obesity, diabetes, chronic pulmonary, renal failure, liver disease, heart disease, fluid electrolyte disorders, alcohol abuse, and anemia were assessed using Charlson comorbidity index. The anion gap (AG), hematocrit, concentrations of bicarbonate, creatinine, chloride, glucose, hemoglobin, potassium, sodium, and blood urea nitrogen (BUN), platelet and white blood cell counts, international normalized ratio (INR), prothrombin time (PT), and partial prothrombin time (PTT) were among the laboratory parameters. Acute Physiology Score III (APSIII), Oxford Acute Severity of Illness Score (OASIS), Sequential Organ Failure Assessment (SOFA) score, Angus score, and Simplified Acute Physiology Score II (SAPSII) are among the severity grading systems.

The database was screened for inflammatory variables, which include albumin, total protein, immunoglobulin, absolute neutrophil count, absolute lymphocyte count, absolute basophil count, absolute monocyte count, absolute eosinophil count, platelet count, lactate, ferritin, CRP, glucose, TM (thrombomodulin), complement, and ESR. Based on data availability and relevance to systemic inflammation, the following variables selected for analysis include gender, age, C-reactive protein (CRP), white blood cells (WBC), absolute basophil count (absbasophil), absolute eosinophil count (absesonophil), absolute lymphocyte count (abslymphocyte), absolute monocyte count (absmonocyte), absolute neutrophil count (absneutrophil), platelet count, albumin, globulin, and glucose. Missing data were handled using multiple imputation techniques using “mice” package in R software (version 4.4.2) [30]. Variables with >20% missing data were excluded from the analysis.

### 2.4. Ratio Calculation and Feature Engineering

Various combinations and permutations of the selected inflammatory markers identified and extracted from the database were explored to create potential inflammation ratios that could have a significant impact on the outcome of secondary malignancy. These ratios were calculated and included as potential predictors in the subsequent analyses.

### 2.5. Statistical Analysis

Baseline data are presented as absolute frequencies and percentages for categorical variables and as means and SDs or medians and interquartile ranges for continuous variables. Based on baseline inflammatory variables taken from the MIMIC-IV database (C-reactive protein, WBC, basophils, eosinophils, neutrophils, leukocytes, monocytes, platelet count, glucose, albumin, and globulin), the least absolute shrinkage and selection operator (LASSO) Cox regression model for dimensionality reduction selected the best prognostic features from all available inflammatory and pertinent ratios.

The correlation coefficients of inflammatory prognostic factors with the result of secondary malignancy, regarded as a binary variable, were estimated using a Spearman correlation analysis, where coefficients |R| > 0.4 and *p* < 0.5 were significantly correlated.

Based on this criterion, the relevant ratios contributing to the development of secondary malignancies were selected. The best ratios were then combined, checked for multicollinearity, and subjected Cox proportional hazard assay. The best features were used to identify the prediction equation based on the hazard ratios and *p*-values. The coefficients of the predictors in the prediction equation are used to generate a systemic risk index for each patient entry. The identified risk index identified was combined with the prognostic scores of other variables documented in the database and laboratory biomarkers. The risk index was also compared with standard inflammation indices reported in the literature. These include the following ratios and indices: NLR(N/L-ratio) [31], PLR(P/L-ratio) [31], prognostic nutrition index (PNI)(10 ALB + 0.005 L) [31], systemic immune-inflammation index (SII), P*NLR) [31], ALI (BMI *ALB/NLR) [31], CAR (CRP/ALB ratio) [32], AGR (ALB/GLB ratio) [33], LCR (L/CRP ratio) [34], GNRI (1.489 ALB + [present body weight (PBW)/ideal body weight (IBW)]) [35], and mGNRI (modified GNRI, 1.489 CRP + 41.7 PBW/IBW) [35].

Univariate analysis: To identify variables that were substantially (*p* < 0.05) linked to the emergence of radiation-induced secondary cancers, a univariate Cox regression analysis was used.

Multivariate analysis: Using a forward stepwise selection process, variables that had a significant correlation in the univariate analysis were added to a multivariate Cox regression model. This model identified independent predictors of the risk of secondary malignancy.

Nomogram construction: The independent factors found in the multivariate Cox regression analysis were then used to build a nomogram. Based on the unique characteristics of each patient, this nomogram graphically depicts the likelihood of acquiring radiation-induced secondary cancers.

Validation of nomograms: The following techniques were used to assess the nomogram’s performance: the C-index, or Harrell’s concordance index, to evaluate the nomogram’s capacity for discrimination; the receiver operating characteristic curve’s area under the curve (AUC) to further measure the capacity for discrimination; calibration curve to assess how well observed results and expected probabilities match; and decision curve analysis (DCA) was used to evaluate the net clinical benefit of applying the nomogram in clinical decision-making.

Comparison of radiation modalities: The risk of secondary malignancy (probability of occurrence) predicted by the nomogram was compared between patients treated with high linear energy transfer (HLET) and low linear energy transfer (LLET) radiation modalities.

Software: All statistical analyses were performed using R software (version 4.4.2), utilizing relevant packages such as “survival”, “survminer”, “rms”, “ggplot2”, and “regplot”.

Statistical tests: A number of statistical tests, including Cox proportional hazards models, Mann–Whitney U tests, and T-tests, were used to evaluate the significance of the data, as and where needed. 

**Figure 1:**
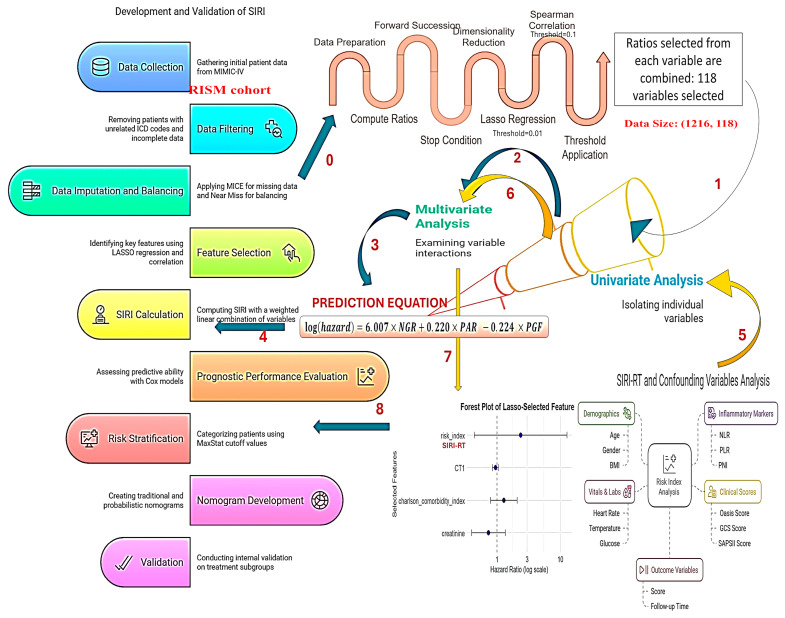
Schematic representation of the methodology and data analysis strategy used for the development and validation of SIRI-RT.

The methodology and data analysis strategy used in this study for development and validation of SIRI-RT has been illustrated in Figure 1.

## 3. Results

This study examined the relationship between secondary malignancy outcomes and inflammation-based prognostic scores in a cohort of 1216 cancer patients exposed to radiation. Approximately 200,000 patients documented in the MIMIC-IV database, cohort with post-radiotherapy secondary cancers defined by ICD-9 and ICD-10 codes, and serum marker levels made up the original dataset.

After filtering out patients with unrelated ICD codes, the analytical cohort consisted of 4433 patients (2585 with and 1848 without secondary malignancy). Secondary malignancies diagnosed post-radiotherapy were determined by comparing the admission time of the patient at the time of secondary malignancy and the chart time when the radiotherapy was given. Furthermore, subjects with specific ICDs such as 8919: video and radio-telemetered EEG monitoring, 8702: other contrast radiogram of brain and skull, and 9205: CVS and haemato-scan and radio-isotope function study, were removed manually, reducing the count for analysis as 1443 (608 with and 835 without secondary malignancy). Missing data imputation was performed using the MICE package, and data balancing was achieved using the Near Miss algorithm, resulting in a final dataset of 1216 patients with an equal number of cases with and without secondary malignancy post-radiotherapy.

### 3.1. Baseline Characteristics

Table 1 presents the baseline characteristics of the study population, along with various hematological, biochemical, and clinical parameters, focusing on the presence or absence of secondary malignancy (SM).

**Figure 2:**
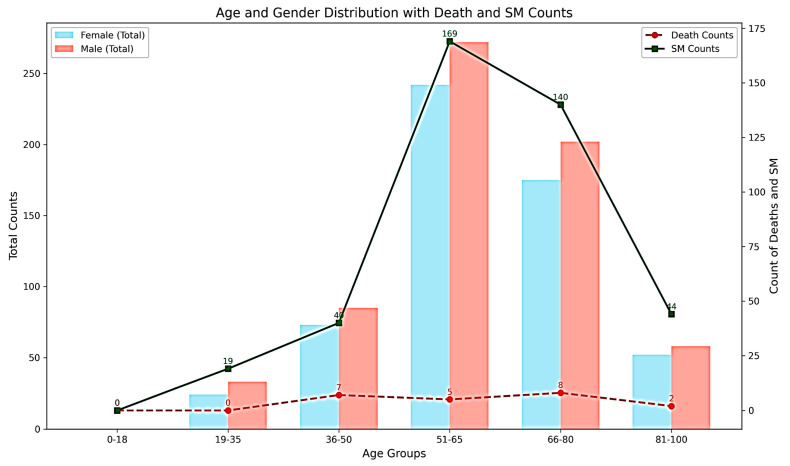
Distribution of age and gender with counts of death and secondary malignancy.

The mean follow-up time was 28.23 months (95% CI: 12–144). The mean age was 62.32 ± 13.6 years, with 51.97% male and 48.02% female patients. No data were recorded for the patients aged <18 years. Secondary malignancies were most frequent in the 51–65 and 66–80 yr old age groups (Figure 2).

**Figure 3:**
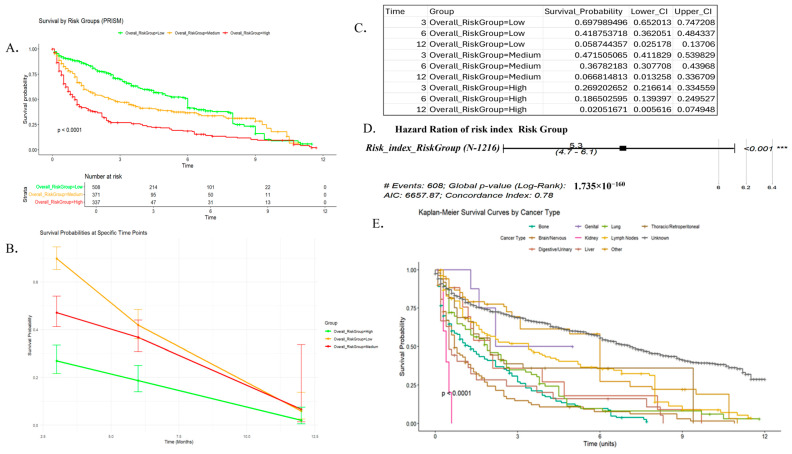
Survival probability of overall risk groups. (**A**) KM plot of overall risk group. (**B**) Survival probability at specific time points. (**C**) Survival probability of three different risk groups at 3, 6 and 12 months. (**D**) Hazard ratio of SIRI-RT. (**E**) KM plot of survival curve by secondary cancers by type post-radiotherapy. Note: *** represents *p* value < 0.001, indicating high significant difference.

The mean time to secondary malignancy was 5.4 months (maximum: 8 years), and the mean time to death in patients with secondary malignancy was approximately 11 months (maximum: 10.4 years). The mean latency of 5.4 months in our cohort may reflect potential misclassification (e.g., recurrence or metastasis misidentified as secondary malignancies), or the inclusion of rapid-onset chemotherapy-related leukemias or MDS. Importantly, these early appearing lesions at secondary sites indicate rapid induction of radiation-induced secondaries caused by enhanced tumor cell migration. The different systemic inflammatory ratios are also calculated and recorded for the cohort studied. Furthermore, the KM survival curve suggests that patients with “unknown” secondary tumor sites (perhaps a heterogeneous group) experience relatively better survival outcomes in this dataset. Brain/nervous tumors have the most pronounced early drop, indicating a very poor prognosis, followed by digestive/urinary and lung tumors with relatively poor survival. Kidney, bone, and lymph node tumors show moderate survival. Genital, thoracic/retroperitoneal, and others tumors do somewhat better than the moderate group (Figure 3).

### 3.2. Development of the Serum Inflammation-Based Risk Index (SIRI-RT) as Prognostic Score

**Figure 4:**
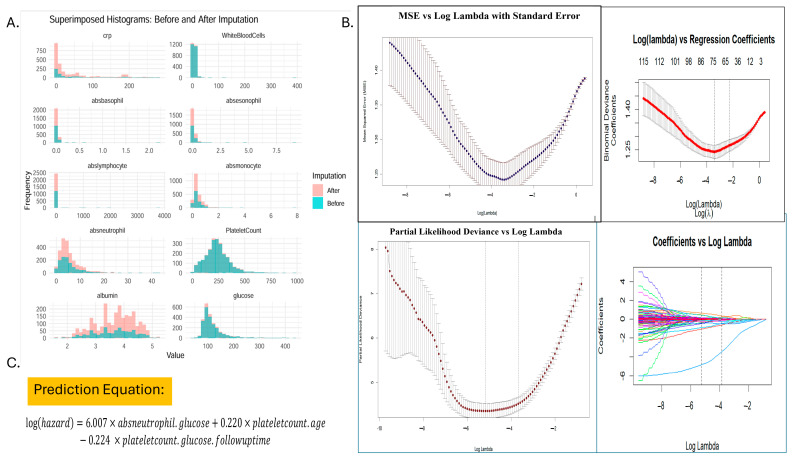
(**A**) Before and after missing value imputation by MICE-package in R software (version 4.4.2). (**B**) The least absolute shrinkage and selection operator (LASSO) Cox regression model screening parameters. (**C**) Prediction equation generated used as SIRI-RT (risk index).

The ratios were computed sequentially using all eleven variables, starting with single-variable ratios and progressively incorporating two, three, or more variables at a time in a forward succession. This process was continued until no further integral values were obtained from the computed ratios. Feature selection using LASSO regression and Spearman’s correlation identified 118 features for further analysis of the optimal λmin = 0.0052 and λ1se = 0.021. The final selected features from the integration of lasso and spearman included 12 variables, which were finally reduced to 3 variables after univariate analysis to generate a prediction equation and hence prognostic score as SIRI (Figure 4).

### 3.3. Prognostic Performance of SIRI-RT—SIRI for Radiotherapy-Induced Secondary Tumors

Univariate Cox proportional hazards analysis of these 12 ratios revealed that absneutrophil/glucose (hazard ratio [HR] = 234.6) was the strongest risk factor, followed by plateletcount/age (HR = 1.1265), which moderately increased the risk with age and platelet count. However, PlateletCount/glucose*followuptime (HR = 0.8571) showed a protective effect, wherein a longer follow-up with stable glucose levels might improve survival. A multivariate Cox model incorporating these three features demonstrated excellent predictive ability (concordance index = 0.852) and was statistically significant (likelihood ratio test, *p* < 2 × 10^−16^; Wald Test, *p* < 2 × 10^−16^; score (log-rank) test, *p* < 2 × 10^−16^). The multivariate model yielded the following prediction equation for the log hazard:

$$\log\left(hazard\right)=6.007\times absneutrophil.glucose+0.220\times plateletcount.age-\;0.224\times plateletcount.glucose.followuptime$$

Further rewritten as follows:

log⁡(hazard)=6.007×NGR+0.220×PAR−0.224×PGF 

**Figure 5:**
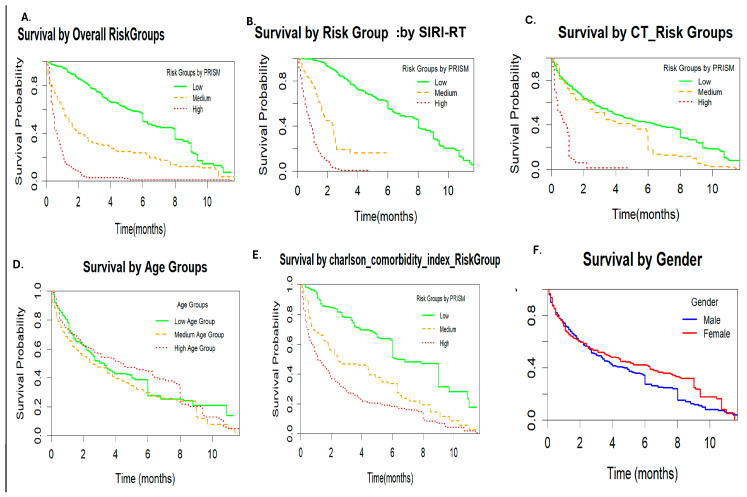
KM plots showing survival difference generated using SIRI-RT scores by different predictor variables. (**A**) Overall risk group; (**B**) risk index-based risk group; (**C**) chemotherapy risk group; (**D**) age; (**E**) Charlson comorbidity index risk group; (**F**) gender risk group.

Kaplan–Meier curves showing survival differences based on SIRI-RT scores are shown in Figure 5.

The coefficients of the generated prediction equation were used to compute the prognostic score as the SIRI-RT:

SIRI=β1X1+β2X2+……….+βpXp

**Figure 6:**
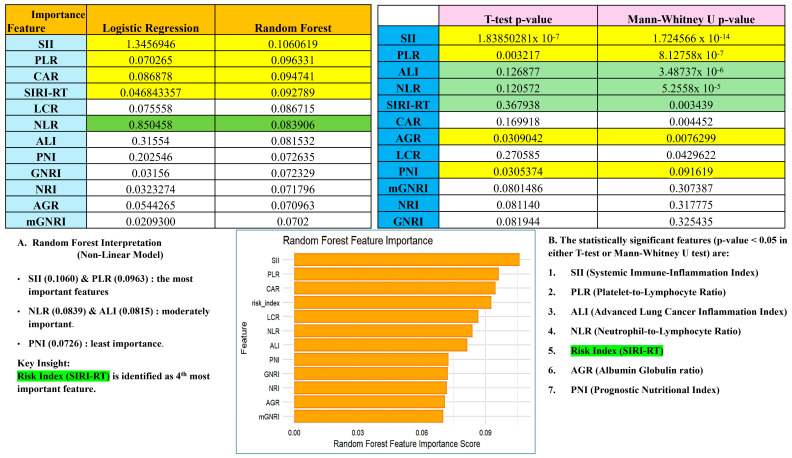
Comparative evaluation of SIRI-RT with the standard-inflammatory indices (as per the literature) for diagnosis of different cancer types. (**A**) Interpretation using Random Forest; (**B**) statistical significance using T-test and Mann–Whitney U-test.

The SIRI generated was then combined with other clinical parameters to identify and evaluate the impact of confounding variables extracted from the database. The confounders included variables from demographics (age, gender, BMI, weight, height), standard inflammatory markers (NLR, PLR, PNI, SII, ALI, CAR, AGR, LCR, GNRI, mGNRI, NRI: computed manually on the dataset), clinical scores: (oasis, gcs_score, sapsii, sepsis3, sirs, apsiii), vitals and labs (heartrate, mbp, resp-rate, temperature, spo2, inr, pt, ptt, hematocrit, hemoglobin, anion-gap, bicarbonate, bun, calcium, chloride, creatinine, glucose, sodium, potassium, Charlson’s comorbidity index), and outcome variables (score, follow-up time). Furthermore, we also compared the prognostic risk index generated in our study with known inflammatory indices in the literature under different conditions (Figure 6), although none of them was found to significantly affect the outcome of secondary malignancy post-radiation.

**Figure 7:**
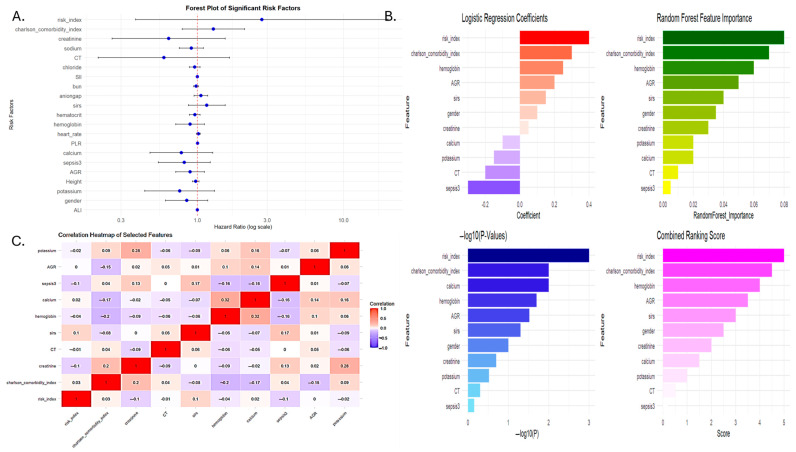
(**A**) Forest plot using univariate analysis. (**B**) Comparative significance of features found after multivariate analysis Cox analysis for the outcome of RISM using logarithmic regression, Random Forest feature, and −logP values. Selection of best features using univariate and penalized Cox regression. (**C**) Heatmap correlation of significant features.

A penalized Cox-regression model (Table 2 using LASSO at (λ1se~0.05642)) further refined the predictor set, selecting four variables: SIRI-RT, Charlson comorbidity index, chemotherapy treatment, and creatinine. This model achieved a C-index of 0.899. This was confirmed by univariate analysis and by studying the association of different clinical and biochemical variables with the outcome of RISM (radiation induced secondary malignancy) (Figure 7).

A final multivariate Cox model (Table 3), incorporating these four variables, showed excellent performance (C-index = 0.897) and high statistical significance (likelihood ratio, Wald, and log-rank tests, all *p* < 2 × 10^−16^).

**Figure 8:**
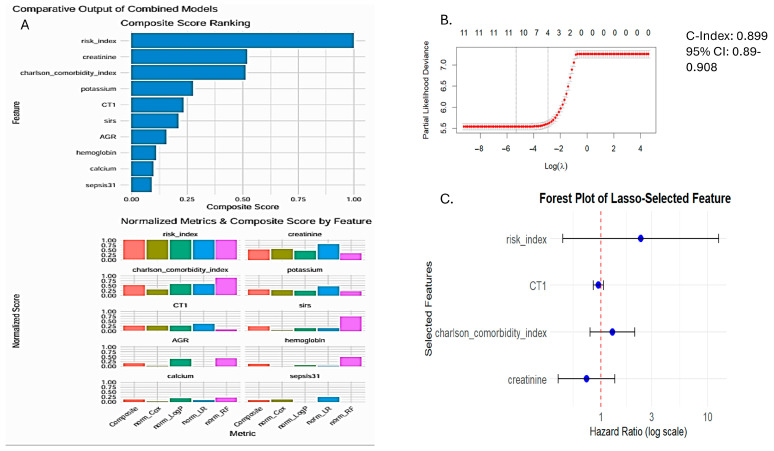
(**A**) Comparing different models to select best features based on composite score generated using normalized metrics of Cox, log P, logarithmic regression, and Random Forest. (**B**) Feature selection using the Cox-penalized model using optimum lambda: 0.00498. (**C**) Forest-plot of features selected using the Cox-penalized model.

The hazard ratios for this final model were SIRI-RT (HR = 3.03), chemotherapy (HR = 1.97), Charlson comorbidity index (HR = 1.47), and creatinine (HR = 0.48). Interestingly, the risk index and Charlson comorbidity index showed the strongest association with survival, both in terms of HR and concordance. Chemotherapy had a significant association but lower concordance, indicating that it might not be as reliable for prediction alone. Creatinine was found to have a protective effect (HR < 1), although the concordance was moderate (Figure 7 and Figure 8). 

Analysis of relative risk (RR) and excess relative risk (ERR) further confirmed their role as confounders in the risk of secondary malignancy. The Charlson comorbidity index continues to be a robust and independent indicator of secondary malignancy risk (RR > 1), underscoring the clinical importance of comorbidity in cancer prognosis and the necessity of incorporating it into risk assessment and treatment strategies. Conversely, creatinine levels and chemotherapy were associated with a reduced risk of secondary malignancy (RR < 1), and this protective effect was enhanced after adjusting for confounders. This indicates that these factors might play a protective role, although further research is needed to confirm these findings and to explore the underlying mechanisms. It is crucial to ensure that the estimated risk of secondary malignancy accurately reflects the true association. This leads to improved risk group prediction, ultimately aiding better clinical decision-making and public health interventions. Therefore, we employed the Axelson indirect adjustment method to correct for factors (e.g., genetic predispositions, lifestyle factors, etc.) that, when not fully measured or adjusted for as confounders, can influence the outcome independently of radiation exposure and introduce bias in the relative risk estimates [36,37]. Interestingly, the risk index (SIRI-RT) developed in our study initially indicated a slightly elevated risk of secondary malignancy (RR > 1), but after adjusting for the hypothetical confounders, it became protective (RR < 1) (using the Axelson method). This finding emphasizes the importance of considering and adjusting for potential confounders, which should be validated in future epidemiological studies.

### 3.4. Risk Stratification

**Figure 9:**
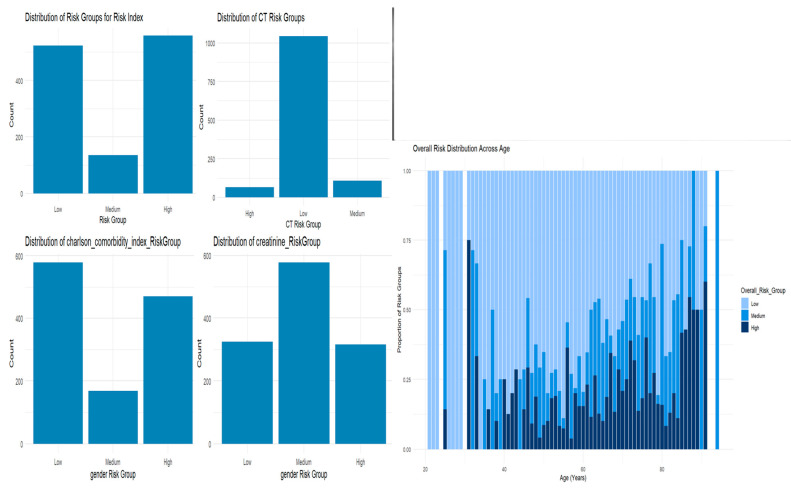
Distribution of risk groups across age and different predictor variables: risk index, chemotherapy, Charlson comorbidity index, and creatinine.

Risk stratification using MaxStat (R software; version 4.4.2), identified cutoff values for the serum inflammatory risk index, Charlson comorbidity index, chemotherapy treatment, and creatinine, allowing for the categorization of patients into three risk categories: low, medium, and high (Figure 9).

The mapped risk group category for each variable was then aggregated to compute the overall risk score. The overall risk score was used to compute the overall risk group for each patient who was exposed to radiation. The log-rank test was used to compare survival distributions across groups for the overall risk group (low, medium, high) from the Kaplan–Meier analysis (Figure 9).

**Figure 10:**
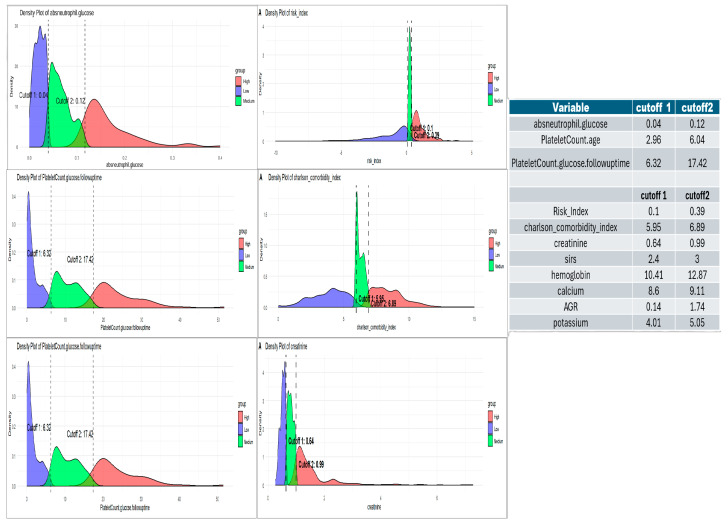
Cut-offs for defining low-, medium-, and high-risk groups for selected inflammatory ratio combination in SIRI-RT and features used in generating the nomogram.

Clear stratification (especially the sharp contrast between low and high risk) indicates that the risk model is strong and effective at separating patient survival probabilities. The overall risk distribution across different age groups clearly showed that patients aged >75 years were in the high-risk group (Figure 10). The log-rank test compares survival distributions between groups for the Kaplan–Meier analysis’s overall risk groupings (low, medium, and high). A Chi-squared statistic of 572 with two degrees of freedom and a *p*-value < 2 × 10^−16^ confirms that the survival curves are not identical.

### 3.5. SURVIVAL Analysis and Progression-Free Survival (PFS)

The Kaplan–Meier plot clearly showed that risk stratification (SIRI-RT, CT, and Charlson comorbidity index) significantly influenced the survival probabilities. The high-risk group consistently showed poorer survival.

**Figure 11:**
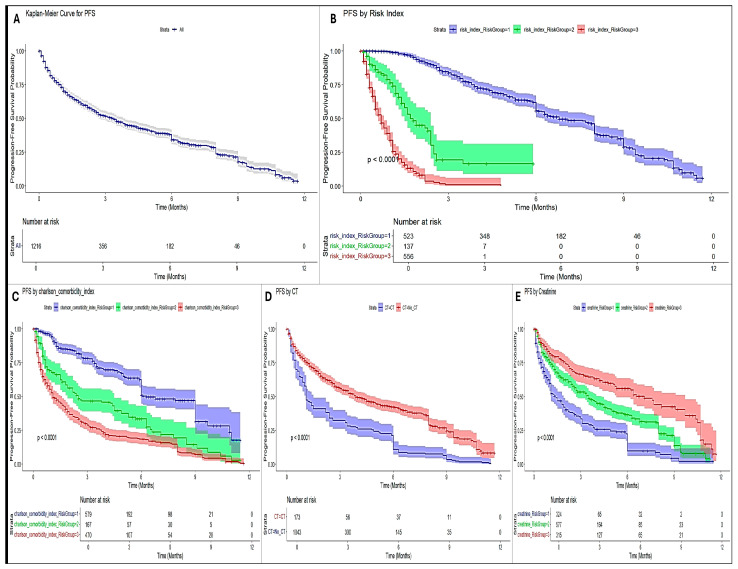
KM plots for PFSs of different predictor variables. (**A**) Overall; (**B**) risk index (SIRI-RT); (**C**) Charlson comorbidity index; (**D**) chemotherapy; (**E**) creatinine.

Age had a modest effect, while sex showed minor differences in survival. This has been validated by the log-rank test showing a Chisq of 96.2 with 2 degrees of freedom, *p* ≤ 2 × 10^−16^ (Figure 5). Furthermore, to predict the onset of post-radiotherapy more precisely and sensitively by avoiding the confounding influences inherent in overall survival (OS) and the composite nature of disease-free survival (DFS), checking the impact of predictors on progression-free survival can be valuable. Chemotherapy was associated with a worse PFS in this cohort. However, creatinine levels, Charlson’s comorbidity index, and risk index (SIRI-RT) effectively stratified patients, with significant differences in PFS. It is notable that SIRI-RT seems to have the most substantial impact on PFS, with clear separation between risk groups, with high HR (5.35; 95% CI (4.67–6.12); *p* < 2 × 10^−16^) and excellent concordance (0.784), followed by Charlson comorbidity index, which is a significant but moderately strong predictor (*p* < 2 × 10^−16^; c-index:0.699; HR:1.97; 95% CI (1.79–2.17)). Chemotherapy was associated with the worst PFS, but had weak predictive ability on its own (c-index: 0.561; HR: 2.12; 95% CI: (1.78–2.53); *p* < 2 × 10^−16^) (Figure 11).

A clear decline in survival probability is observed from low- to high-risk groups across all time points. The median survival time for the RISM cohort from MIMIC-IV is observed to be 2.4 months, with a narrow 95% CI ranging from 2.2 to 2.6 months, suggesting a precise estimate.

At 12 months, survival for high-risk patients was almost negligible (2%) compared to that for low-risk patients (~5.9%), highlighting the predictive value of risk stratification. All groups showed declining survival probabilities over time, as expected in the survival analysis, but confidence intervals widened over time because fewer subjects remained under observation over longer durations, leading to increased variability (Figure 2).

### 3.6. Nomogram Generation and Validation

**Figure 12:**
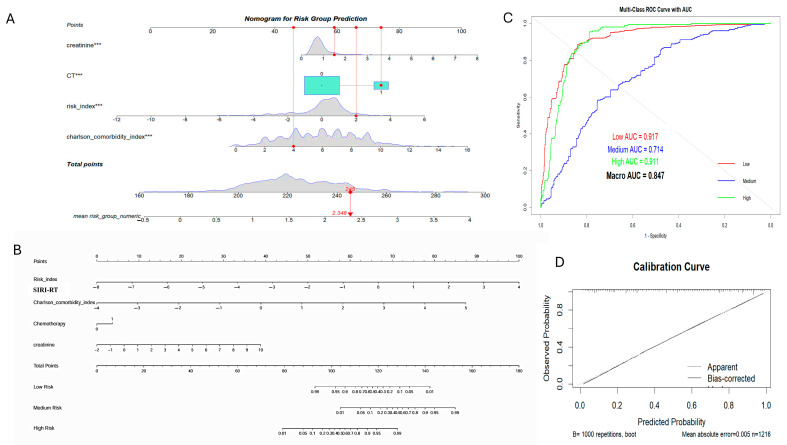
Generation and validation of nomogram for risk group prediction. (**A**) Nomogram showing mean risk group numeric using regplot; (**B**) nomogram showing risk group stratification separately; (**C**) AUC-ROC curve showing AUC for the prediction of the low-risk group: 0.91, medium-risk group as 0.714, high-risk group 0.91; overall AUC: 0.947; (**D**) calibration curve.

Two nomograms were developed: a traditional nomogram for quick risk assessment in a clinical setting, and a nomogram displaying survival probabilities at 1, 3, and 5 years (Figure 12).

**Figure 13:**
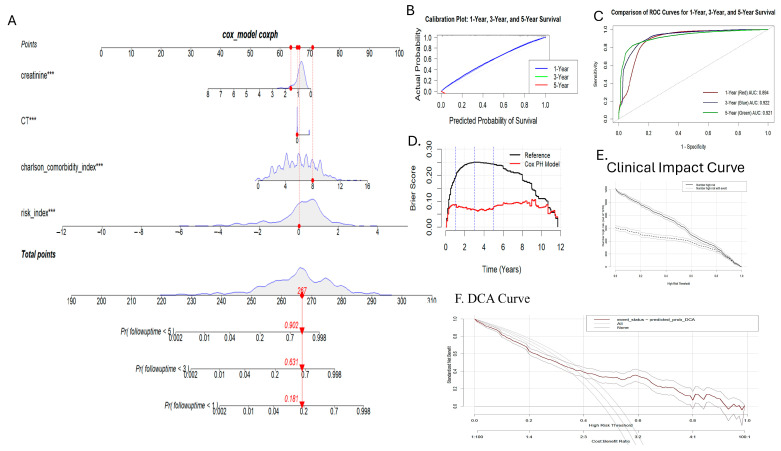
Generation and validation of nomogram to predict survival probabilities of radiation-induced secondary malignancy (RISM) at different time points (1, 3, and 5 years). (**A**) Nomogram; (**B**) calibration plot for 1, 3, and 5 years; (**C**) comparison of ROC for different time points with AUC of 1 yr: 0.89, 3 yr: 0.92, and 5 yr: 0.91; (**D**) Brier score; (**E**) clinical impact curve (CIC); (**F**) DCA: decision curve analysis. Note: *** represents *p* value < 0.001, indicating high significant difference.

The nomograms were validated using ordinal logistic regression (polr) and bootstrapping, which demonstrated strong discrimination, calibration, and minimal overfitting. The C-index (~0.76) and Gini coefficient (~3.33) suggested that the model not only effectively distinguished between the risk groups, but also showed high accuracy with balanced sensitivity/specificity (~72% at 95% CI (0.7255, 0.7749) and significant *p* < 2 × 10^−16^) (Figure 12). Interestingly, the specificity of the nomogram model was higher than the sensitivity, ensuring that none of the high-risk and medium-risk groups were misclassified. The model, when tested with the random forest algorithm, yielded a prediction of comparable sensitivity and specificity with an accuracy 87.99 at 95% CI ((0.8603, 0.8977) and *p* < 2 × 10^−16^). However, the nomogram was generated using regplot package maps directly to predict survival probabilities at different time points (1, 3, and 5 years) by fitting the Cox proportional model. A C-index of 0.897 and Brier score of 0.076 explained the predictive accuracy of the survival model. Clinical impact curves (CIC) and decision curve analysis (DCA) have provided additional evidence for the clinical usefulness of nomograms. The AUC of the nomogram was found out to be 0.894, 0.922, and 0.921 at 1, 3, and 5 years, respectively (Figure 13).

### 3.7. Subgroup Analysis and Validation

Specific patients exposed to different types of radiation treatment were selected and filtered based on the long-title description of the procedure ICD codes. Internal validation of the risk prediction model for secondary malignancy was applied to four subgroups of patients: those treated with high-LET radiation (HLET), low-LET radiation (LLET), surgery, and brachytherapy. This not only validates but also demonstrates the application of a risk index (SIRI-RT) and the subsequent evaluation of the nomogram model performance using a risk prediction function. This function enables clinicians to input patient-specific laboratory values and calculate the prognostic score (SIRI-RT) based on a previously established prediction equation using different ratio combinations of inflammatory parameters. The function then categorizes patients into low-, medium-, and high-risk groups based on the cut-off values for the SIRI-RT, Charlson comorbidity index, chemotherapy, and creatinine. Finally, we calculated a predicted risk score (sum of individual risk scores) and assigned an overall predicted risk group based on predefined cut-off values. Hence, this function can be implemented as an R-script function, allowing for rapid and clinically practical prediction of patient risk, thereby facilitating personalized decision-making in clinical settings.

**Figure 14:**
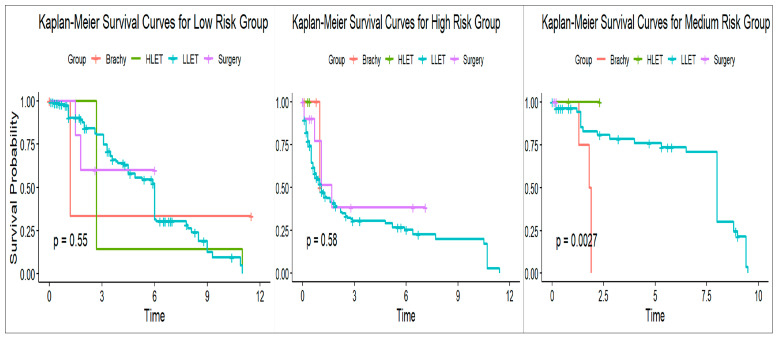
KM–survival curves for subset population after prediction as internal validation for low-, medium-, and high-risk groups.

Model performance was evaluated using a confusion matrix that compares the predicted risk group with an overall risk group (generated in the entire RISM cohort from the MIMIC-IV database). The accuracy of prediction varied across the subgroups, with the LLET group showing the highest accuracy (~96%), whereas the surgery and brachytherapy groups showed lower accuracies (~90% and 93%, respectively). The HLET group’s accuracy was observed to be 100%, which can potentially be attributed to the very limited available data of patients being exposed to high-LET radiotherapy. The Kaplan–Meyer survival plot for high LET, low LET, brachytherapy, and radiosurgery for the RISM cohort in MIMIC-IV shows better survival for HLET, radiosurgery, and brachytherapy as compared to LLET, despite the limitation of sample size (Figure 14).

Furthermore, an additional validation process is required to be performed using external real-world data to test the model, as logic might fail in real-world scenarios.

## 4. Discussion

This study investigates the association between inflammation-based prognostic scores and secondary malignancy outcomes in patients exposed to radiation therapy. By utilizing the MIMIC-IV database and incorporating various clinical and laboratory parameters, this study demonstrates the power of big data analytics in oncology research.

In light of the existing scientific literature, this study builds upon previous work on inflammation-based prognostic scores in cancer [38,39,40,41]. It extends the application of such scores to the specific context of radiation-induced secondary malignancies, thereby addressing a critical gap in the field.

Radiation-induced secondary malignancies are a significant concern for cancer survivors, with the risk dependent on factors such as patient age, genetic predisposition, irradiated tissue volume, and radiation dose [42]. Studies have shown an increased risk of secondary malignancies in patients treated with radiation therapy compared to those without, particularly in those with breast cancer [43]. Post-radiotherapy inflammation, triggered by reactive oxygen species, infiltrating immune cells, and pro-inflammatory cytokines, leads to progressive tissue damage, making the cellular microenvironment conducive to secondary carcinogenesis [44]. The inflammatory state in patients with cancer can chronically alter the microenvironment to drive secondary carcinogenesis [45]. However, the slow and progressive nature of post-radiotherapy inflammation can worsen patient prognosis [46]. After radiotherapy, inflammatory cytokines initiate inflammatory loops that can have systemic consequences contributing to carcinogenesis [47]. Hence, serum inflammation is not just indicative of risk, but can be a good predictive tool for better treatment response and survival outcomes [48]. For both patients and clinicians, knowing the period during which a patient remains free from any new malignant progression is important for evaluating both the efficacy and safety of the treatment. The influence of filtered predictors on PFS and OS serves as a direct indicator of the period during which the patient is free from adverse secondary effects, specifically defined to include only relevant outcomes of progression, such as secondary cancers.

Therefore, it is important to highlight the serological characteristics of tumor progression for further risk stratification and individualized treatment. Using ratios of inflammatory variables to develop prognostic scores can be highly valuable because, apart from improving prognostic accuracy, it can help capture the balance between the components of the immune response and reduce some of the variability inherent in absolute measurements [49]. When developing prognostic scores with multiple variables, using forward ratios imparts directional consistency with enhanced interpretability, stability, and robustness [50].

We developed a Serum Inflammation-Based Risk Index (SIRI-RT) as a prognostic score for predicting radiotherapy-induced secondary tumors. This tool combines inflammatory markers with clinical parameters to provide a comprehensive risk assessment. The present investigation was conducted on a retrospective cohort from the MIMIC-IV database, in which serum values of inflammatory parameters were subjected to all combinations of forward ratios. With an average follow-up time of 2.35 years post-radiotherapy and data on long-term outcomes, this study offers valuable information on the temporal dynamics of secondary malignancy development post-radiotherapy.

To reduce the interference between variables, we used LASSO regression and Spearman’s correlation analysis to reduce dimensionality while considering collinearity and correlation between various ratios and indicators. Sensitivity analysis was performed by varying the feature selection criteria, including Spearman’s correlation thresholds and regularization parameters (alpha and lambda) in the LASSO regression. Features consistently selected across these methods were subjected to further validation using univariate and multivariate Cox regression analyses. This robust approach ensured that the features retained in the final model demonstrated stable and significant predictive value, thus justifying their inclusion. The final combination of ratios when combined and subjected to univariate analysis generated a prediction equation using the coefficient values. The final selected variables based on HR, *p*-value, and likelihood ratio helped us in generating an index “SIRI-RT”, which incorporates absneutrophil/glucose (NGR), PlateletCount/age (PAR), and PlateletCount/glucose*followuptime (PGF), which can be used as a therapeutically useful risk stratification tool. However, risk assessment is always affected by confounders in radiation carcinogenesis, which influences treatment outcomes [51]. Hence, when combined with demographic, clinical, vital, and other laboratory factors, SIRI-RT was found to be significantly affected by comorbidities identified by the Charlson comorbidity index. In addition to the ability of SIRI-RT to predict RISM, chemotherapy and electrolyte balance are other factors that have been strongly linked, but with a decreased predictive potential. The superior performance of SIRI-RT, demonstrated by a high concordance index (0.897) and AUC values exceeding 0.9 in tested cohorts, underlines the significance of integrating inflammatory markers into prognostic models for secondary malignancies. The substantial hazard ratios associated with SIRI-RT components, such as the increased risk linked to a high neutrophil–glucose ratio, lend biological plausibility to the findings and suggest a critical role for systemic inflammation in the development of post-radiation therapy secondary malignancies. However, the protective effect observed with Plateletcount/glucose*followuptime necessitates further exploration to elucidate the underlying mechanisms. SIRI-RT was combined not only with demographic, clinical, vital, and other laboratory parameters, but also with the standard inflammatory indices used in the literature to determine their relative impact on the outcomes of RISMs. However, univariate analysis revealed that out of 21 variables extracted from the database, only 10 had the most significant impact on post-radiotherapy outcomes. In addition to chemotherapy and comorbidities, creatinine (HR: 0.63), hemoglobin (HR: 0.89), sepsis3 (HR: 0.81), and electrolytes (sodium, potassium, and calcium) were found to impact the outcomes, but were protective (HR < 0.9 and *p* < 0.05). Although the multicollinearity of the variables was not statistically significant (VIF < 2), we selected the best features. Hence, SIRI-RT, along with other clinical factors (Charlson comorbidity index, chemotherapy treatment, and creatinine levels), was used to effectively stratify patients into low-, medium-, and high-risk groups. Multivariate Cox analysis confirmed the predictors with a c-index of 0.897. This stratification can guide personalized treatment decisions and follow-up strategies.

The protective association of creatinine and chemotherapy was also observed with RR and ERR values, in contrast to SIRI-RT and the comorbidity index, which doubled the hazard. Hence, this clearly highlights the importance of inflammatory markers, comorbidities, and treatment-related factors in predicting secondary malignancies, providing insights into the potential mechanisms and targets for intervention. The aggregated risk score was generated by assigning groups to the predictors using the cutoff points generated using the MAXSTAT package in R software (version 4.4.2) to stratify the overall risk groups.

The development of nomograms for quick risk assessment and survival probability estimation at 1, 3, and 5 years enhances the clinical utility of these findings. These tools can be easily integrated into clinical practice for rapid patient evaluation, providing time-specific survival probabilities, aiding treatment planning, and shared decision-making with patients. Curiously, we endeavored to develop a nomogram aimed at predicting risk categories, which involves a more probabilistic rather than deterministic evaluation for RISMs as chronic conditions. The validation of these nomograms using ordinal logistic regression and bootstrapping coupled with supportive results from decision curve analysis (DCA) and clinical impact curves (CIC) affirms their reliability and clinical value. The DCA revealed that the model consistently outperformed all and none of the strategies across various risk thresholds, indicating its practical applicability in clinical decision-making. The CIC offers insights into the balance between over- and under-treatment, guiding clinicians in the appropriate application of the nomogram.

The risk stratification model and nomograms developed in this study can help design more personalized treatment approaches, potentially improving patient outcomes and quality of life [52]. Individuals at elevated risk may undergo more rigorous monitoring, be evaluated for alternative treatment options, or participate in preventive research. In contrast, those at lower risk might be spared from unnecessary intensive monitoring or interventions. This study further conducted internal validation and subgroup analyses based on radiation type (high LET, low LET, and brachytherapy), demonstrating the robustness of the model across different treatment modalities.

The findings of this study align with the growing evidence that chronic inflammation plays a role in the development and spread of cancer [53]. The predictive value of inflammatory markers for secondary malignancies reinforces the importance of monitoring and potentially modulating inflammatory processes in cancer patients undergoing radiotherapy [54]. By identifying high-risk individuals, healthcare resources can be used more effectively with monitoring, appropriate preventive measures, and treatment resources, ultimately improving overall healthcare outcomes and reducing costs.

Furthermore, the development and validation of practical clinical tools (nomograms and risk stratification models) [55] address the need to translate complex statistical models into user-friendly instruments for clinicians. This approach enhances the potential for an immediate clinical impact and improves patient care. It is important to emphasize that our current findings are supported by robust internal validation; however, additional prospective external validation is essential before the model can be widely adopted in clinical environments. Although we lacked an external dataset for validation in this study, we utilized comprehensive internal validation techniques, such as cross-validation and bootstrapping, to assess the model’s performance and minimize the risk of overfitting [56]. These internal validation methods provided strong preliminary evidence for the reliability of the model within the MIMIC-IV dataset. However, we recognize that internal validation alone does not fully address potential biases or guarantee that the model will generalize across various patient populations. Therefore, prospective external validation in future research would be necessary [57], applying SIRI-RT to external datasets from varied clinical settings to verify its generalizability and enhance its predictive accuracy.

While acknowledging the study’s limitations is crucial for responsible interpretation, these challenges present opportunities for future research. Prospective studies with more comprehensive data collection and standardized protocols could address some of the current limitations, providing more robust evidence for the clinical utility of SIRI-RT and nomograms. Additionally, multi-center collaborations and international registries could help validate the findings across diverse patient populations and healthcare settings.

The analysis revealed an incidence of 4.28% cases of RISMs using SIRI-RT with Charlson’s comorbidity index, chemotherapy, and creatinine levels as important confounding risk factors, where chemotherapy and creatinine are more protective than hazardous in nature. The findings presented herein warrant further investigation into the effects of diverse chemotherapy protocols and creatinine concentrations on the development of radiation-induced secondary malignancies (RISMs). Such research could potentially inform more individualized treatment approaches and tailor radiation therapy regimens to minimize the risk of secondary cancers while maintaining therapeutic efficacy. By examining a broader spectrum of chemotherapeutic agents and their interactions with radiation, researchers may uncover specific combinations that either exacerbate or mitigate RISM risk, leading to more refined treatment guidelines. Subsequent studies may explain the protective mechanisms associated with certain risk factors and reveal novel strategies for mitigating radiation-induced adverse effects. For instance, the observed protective effect of higher creatinine levels merits further exploration to understand the underlying physiological processes. This knowledge could potentially be leveraged to develop pharmacological interventions that mimic or enhance these protective mechanisms, thereby reducing the RISM incidence in high-risk patients. Longitudinal investigations tracking patients over extended durations could yield valuable insights into the long-term consequences of radiation therapy and progression of RISMs. Such studies would provide a more comprehensive understanding of the temporal dynamics of RISM development, including potential latency periods and factors influencing tumor growth rates. This information could be crucial for establishing evidence-based follow-up protocols and early detection strategies for cancer survivors who have undergone radiation-therapy. Furthermore, the integration of advanced imaging modalities and molecular profiling techniques in future research endeavors could enhance our understanding of the underlying biological processes and potentially identify early biomarkers for RISM development. High-resolution imaging techniques, such as functional MRI or PET-CT, could offer new perspectives on tissue changes following radiation exposure. Concurrently, genomic and proteomic analyses of irradiated tissues and emerging secondary tumors may reveal specific molecular signatures associated with RISM risk or progression.

The incorporation of artificial intelligence and machine learning algorithms in analyzing large-scale patient data can uncover previously unrecognized patterns or risk factors for RISM development [58]. These computational approaches could potentially lead to the creation of predictive models that assess individual patient risks more accurately, enabling personalized surveillance and intervention strategies. Additionally, investigating the potential synergistic effects between radiation therapy and emerging cancer treatments, such as immunotherapy or targeted molecular therapies, could provide insights into novel combination strategies that not only enhance primary tumor control, but also reduce the risk of secondary malignancies. This research direction could pave the way for more comprehensive and safe cancer treatment paradigms. Investigating how lifestyle elements like nutrition, physical activity, and stress control affect the risk of RISM after radiation therapy could provide crucial insights for crafting comprehensive cancer survivorship strategies. Understanding how these modifiable factors interact with radiation-induced tissue changes may lead to evidence-based recommendations for post-treatment patient care and long-term health maintenance.

Recommended clinical pathway for integrating SIRI-RT into oncology workflow:

1. Patient selection and baseline assessments:
  - Initial evaluation: At the time of diagnosis and before initiating radiotherapy, patients should undergo a comprehensive evaluation including clinical, laboratory, and radiological assessments.
  - SIRI-RT calculation: Incorporate SIRI-RT, which uses routinely collected clinical parameters, to generate a baseline risk index.
2. Risk stratification:
  - Classification into Risk Groups: Based on the SIRI-RT score, patients are stratified into low-, medium-, and high-risk groups for adverse outcomes such as secondary malignancies.
  - Multidisciplinary Review: During tumor board meetings, specialists such as oncologists and radiologists conduct a multidisciplinary review, where they assess risk stratification by combining these insights with other clinical information.
3. Tailored treatment planning:
  - High-risk patients: For individuals classified as high-risk, consider implementing changes such as the following:
    - More frequent monitoring and follow-up appointments;
    - Alterations in the dosage or methods of radiotherapy delivery;
    - Incorporation of additional systemic treatments to reduce risk.
  - Low-risk patients: Standard radiotherapy protocols may be maintained with routine follow-up.
  - Intermediate-risk patients: Use a balanced approach, potentially incorporating additional diagnostic imaging or biomarkers to further refine risk assessment.
4. Ongoing monitoring and reassessment:
  - Dynamic risk evaluation: Re-assess SIRI-RT at defined intervals during and after radiotherapy to monitor changes in risk profile.
  - Adjusting follow-up protocols: Based on changes in SIRI-RT scores, adjust follow-up intensity and imaging schedules to promptly detect any treatment-related adverse effects.
5. Integration into clinical decision support systems:
  - Electronic Health Records (EHR) integration: Embed SIRI-RT within the EHR to automatically calculate risk scores and flag high-risk patients.
  - Decision support alerts: Provide real-time decision support for clinicians, prompting re-assessment or intervention when significant changes in SIRI-RT are detected.
6. Continuous quality improvement:
  - Outcome tracking: Registries can be utilized to track patient outcomes relative to their SIRI-RT scores.
  - Model refinement: The risk model based on real-world data and outcomes to improve its predictive accuracy and clinical utility can be periodically reviewed and updated.

By adopting this approach, oncologists and radiologists can successfully integrate SIRI-RT into the clinical setting, enabling more tailored treatment plans and proactive care of patients susceptible to radiation-related complications. We believe that this approach not only enhances patient care, but also supports ongoing research and quality improvement in radiation oncology.

## 5. Conclusions

This study offers strong evidence supporting the clinical usefulness of an inflammation-based prognostic score for predicting the outcomes of secondary malignancies in patients who have undergone radiation exposure. The readily available SIRI-RT and accompanying nomograms offer valuable tools for risk stratification, enabling personalized treatment strategies and improved patient management. It can be helpful in improving our understanding of how inflammation, cancer therapy, and long-term oncological outcomes interact. Further research should focus on validating these findings in independent cohorts, exploring the underlying biological mechanisms, as well as the role of other inflammatory markers and interactions between inflammation and other risk factors, to further refine and optimize SIRI-RT for improved clinical outcomes. Mechanistic studies are crucial for understanding the biological pathways linking inflammation to the development of secondary malignancy. The subgroup analysis comparing different radiation modalities further suggests avenues for tailoring risk prediction and management strategies based on radiation type.

## Figures and Tables

**Table 1:** Baseline characteristics.

**Cancer Patients** [Counts (%total pts in MIMIC-IV)]	**42,296** (unique)
	Total
Chemotherapy [Total Counts—in MIMIC-IV]	3797
Surgery [Total Counts—in MIMIC-IV]	59,228
Immunotherapy [Total Counts as treated in MIMIC-IV]	3296
Secondary Malignancy [Total Counts diagnosed in MIMIC-IV]	35,143 (includes total pts with RT and non-RT)
Radiotherapy [Total Counts—in MIMIC-IV]	3851 (with duplicates)
Mean Time Since Radiation Exposure (years)	2.4 (Max: 11.7 y)
**Radiotherapy** [TOTAL RT Cohort-Counts]	1443 (without duplicates)
**Radiotherapy** [TOTAL RT Cohort-Counts used for analysis]	1216
**Radiation-Induced Secondary Malignancy** [RISM-Counts]	608
Age [Mean (sd)]	62.32 y ± 13.6
Male [Counts (%)]	632 (51.97%)
Female [Counts (%)]	584 (48.02%)
**Death Events** [ RISM cohort]	
Male [Counts (%)]	10 (1.5%)
Female [Counts (%)]	12 (2%)
**Secondary Malignancy** [(RISM cohort)]	
Male [Counts (%)]	286 (47.03%)
Female [Counts (%)]	322 (52.96%)
**Subgroups** [TOTAL RT (RISM cohort)]	
High-LET [Counts available in MIMIC-IV]	13 (11)
Low-LET [Counts available in MIMIC-IV]	1122 (502)
Surgery [Counts available in MIMIC-IV]	27 (20)
Brachytherapy [Counts available in MIMIC-IV]	101 (14)
**Radiotherapy Regimens** [TOTAL RT (RISM cohort)]	
Short course [Counts available in MIMIC-IV]	366 (257)
Long course [Counts available in MIMIC-IV]	409 (242)
**RT Treatment Type** [TOTAL RT (RISM cohort)]	
Neoadjuvant [Counts available in MIMIC-IV]	72 (27)
Adjuvant [Counts available in MIMIC-IV]	144 (40)
Definitive [Counts available in MIMIC-IV]	144 (30)
**Dose of Radiation** [TOTAL RT (RISM cohort)]	
0–2 Gy [Counts available in MIMIC-IV]	3 (1)
2–10 Gy [Counts available in MIMIC-IV]	10 (3)
10–20 Gy [Counts available in MIMIC-IV]	19 (3)
20–30 Gy [Counts available in MIMIC-IV]	16 (1)
30–40 Gy [Counts available in MIMIC-IV]	11 (3)
40–50 Gy [Counts available in MIMIC-IV]	17 (8)
50–60 Gy [Counts available in MIMIC-IV]	16 (5)
60–70 Gy [Counts available in MIMIC-IV]	4 (2)
>200 Gy [Counts available in MIMIC-IV]	3 (1)
**Tumor Staging** [TOTAL RT (RISM cohort)]	
TUMOUR (T) [Counts available in MIMIC-IV]	1267 (365)
NODE (N) [Counts available in MIMIC-IV]	967 (264)
METASTASIS (M) [Counts available in MIMIC-IV]	758 (201)
**Primary Condition as per ICD Codes Treated with** RT [RISM Cohort- in MIMIC-IV]	
Other/Non-Cancer or Unclassified [Counts (%)]	523 (43.01%)
Lung Cancer [Counts (%)]	67 (5.5%)
Brain/CNS Cancer [Counts (%)]	61 (5.02%)
Lymphoma [Counts (%)]	52 (4.28%)
Myeloma [Counts (%)]	52 (4.28%)
Leukemia Total (ALL + AML + CLL + CML) [Counts (%)]	48 (20 + 19 + 6 + 1) (3.95%)
Bone Cancer [Counts (%)]	46 (3.78%)
Gynecological Cancer [Counts (%)]	30 (2.47%)
Other Specified Cancers (incl Pancreatic, Liver, Oral, esophageal, Colorectal, Prostate, bladder, etc.) [Counts (%)]	108 (<3%)
**RISM Counts as per ICD Codes Post-Radiotherapy** [RISM Cohort- in MIMIC-IV]	608
Other Cancer Types (Unspecified) [Counts (%)]	273 (44.90%)
Bone Cancer [Counts (%)]	134 (22.04%)
Brain Cancer [Counts (%)]	93 (15.29%)
Lung Cancer [Counts (%)]	77 (12.66%)
Liver Cancer [Counts (%)]	24 (3.95%)
Other Cancers (Rectal, Bladder, Kidney, etc.) [Counts (%)]	7 (1.15%)
**Primary Hematological Conditions as per ICD Codes treated with RT** [RISM Cohort- in MIMIC-IV]	
Myeloma/Plasma Cell Disorder [Counts]	52
Lymphoma—(Other/Hodgkins) [Counts]	49/3
Leukemia (Other/AML/ALL/CML/CLL) [Counts]	7/19/15/6/1
Neutropenia [Counts]	8
Myeloid Neoplasm/MDS/MPN [Counts]	6
Anemia [Counts]	3
Thrombocytopenia [Counts]	1
Pancytopenia [Counts]	1
**Inflammatory Markers** [RISM Cohort—in MIMIC-IV]	
Absolute Neutrophil Count [Mean (sd)] (cells/µL)	71.3 ± 12.2
Absolute Lymphocyte Count [Mean (sd)] (cells/µL)	17.6 ± 10.7
Platelet Count [Mean(sd)] (×10^3^/µL)	229.8 ± 104.86
Albumin(g/dL)-[Mean(sd)]	3.55 ± 0.65
Globulin(g/dL)-[Mean(sd)]	2.7 ± 0.74
Hemoglobin [Mean (sd)] (g/dL)	10.8 ± 1.9
Hematocrit [Mean (sd)] (%)	32.9 ± 5.4
Creatinine [Mean (sd)] (mg/dL)	1 ± 0.8
Sodium [Mean (sd)] (mEq/L)	138 ± 3.28
Potassium [Mean (sd)] (mEq/L)	4.15 ± 0.35
Chloride [Mean (sd)] (mEq/L)	101.74 ± 3.96
Blood Urea Nitrogen (BUN) [Mean (sd)] (mg/dL)	19.83 ± 11.96
Glucose [Mean (sd)] (mg/dL)	120.8 ± 33.22
Bicarbonate [Mean(sd)] (mEq/L)	25.9 ± 2.99
Anion Gap [Mean (sd)] (mEq/L)	13.7 ± 2.08
BMI	79.7 ± 2313
**Vital Signs** [RISM Cohort- in MIMIC-IV]	
heart-rate [Mean(sd)] (bpm)	88.28 ± 14.5
mbp [Mean (sd)] (mmHg)	79.33 ± 9.68
resp_rate [Mean (sd)] (%).	19.3 ± 3.4
temperature [Mean (sd)]	36.8 ± 0.42
sPO2 [Mean (sd)] (%)	96.7 ± 1.87
**Severity Score** [RISM Cohort- in MIMIC-IV]	
Oasis [Mean (sd)]	30.44 ± 7.55
gcs_score [Mean (sd)]	1.9 ± 2.37
sapsii [Mean (sd)]	38.1 ± 13
sepsis3 [Mean (sd)]	0.25 ± 0.436
sirs [Mean (sd)]	2.57 ± 0.83
apsii [Mean (sd)]	43.7 ± 17.9
**Coagulation Parameters** [RISM Cohort- in MIMIC-IV]	
inr [Mean (sd)]	1.28 ± 0.42
pt [Mean (sd)]	14.1 ± 4.29
ptt [Mean (sd)]	34.4 ± 11.68
**Charlson Comorbidity Index** [Mean(sd)] [RISM Cohort- in MIMIC-IV]	4.38 ± 2.88
**Standard Inflammation Ratios** (Literature reported) [calculated in RISM cohort of MIMIC-IV DATABASE]	
NLR [median (IQR)]	6.14(10.6)
PLR (median (IQR)]	274 (378.5)
ALI [median (IQR)]	16.44 (29.11)
SII [median (IQR)]	1333.6 (2490.9)
CAR (median (IQR))	4.89 (24.98)
GNRI [median (IQR)]	−25.24 ± 198.2
mGNRI [median (IQR)]	−1197.15 ± 8378.7
NRI [median (IQR)]	−1285.2 ± 8330.5
AGR [median (IQR)]	1.36 ± 89.3
PNI [median (IQR)]	36 ± 9.9
LCR [median (IQR)]	320.6 ± 19.18

**Table 2:** Lasso Cox-penalized hazard ratios.

Coefficient	Feature	Hazard Ratio
1.065059427	risk_index (SIRI-RT)	2.901011
−0.280998265	CT1 (Chemotherapy)	0.75503
0.318255732	Charlson comorbidity index	1.374728
−0.578412143	Creatinine	0.560788
0.061338537	sirs	1.063259
−0.030315099	Hemoglobin	0.97014
−0.061052613	Calcium	0.940774
−0.140983725	Sepsis31	0.868503
0.044216404	AGR	1.045209
−0.292879789	Potassium	0.746112

**Table 3:** Summary of multivariate Cox proportional hazards model.

Variable	Coefficient (β)	HR (exp(β))	95% CI	*p*-Value	Interpretation
Risk index (SIRI-RT)	1.1089	3.03	2.79–3.30	<2 × 10^−16^ ***	A unit increase in risk_index triples the hazard (~203% higher risk). Strongest risk factor.
Chemotherapy	0.6761	1.97	1.63–2.37	7.39 × 10^−13^ ***	Presence of Chemotherapy nearly doubles the hazard (~97% higher risk).
Charlson comorbidity index	0.3848	1.47	1.41–1.53	<2 × 10^−16^ ***	For each unit increase in the comorbidity score, the hazard increases by 47%.
Creatinine	−0.7246	0.48	0.40–0.58	3.86 × 10^−14^ ***	Higher creatinine is protective, reducing hazard by 52%.

Note: *** represents *p* value <0.001, indicating high significant difference.

## Data Availability

The original data presented in the study are openly available in MIMIC-IV database at URL: https://physionet.org/content/mimiciv/3.1/ (accessed on 12 July 2024; Record ID 63563991) and Ref No.: R01EB030362.

## Abbreviations

The following abbreviations are used in this manuscript:

RISM	Radiation-Induced Secondary Malignancy
AG	anion gap
AGR	Albumin–Globulin ratio
ALI	advanced lung cancer inflammation index
APSIII	acute physiology score III
AUC	area under the curve
BUN	blood urea nitrogen
CAR	CRP_Albumin ratio
CI	confidence interval
CIC	clinical impact curve
CNS	central nervous system
CRP	C-reactive protein
DCA	decision curve analysis
DFS	disease-free survival
GCS score	Glasgow Coma Scale
GNRI	Geriatric Nutrition Risk Index
HLET	high linear energy transfer
HR	hazard ratio
IARC	International Agency for Research on Cancer
ICD	International Classification of Diseases
INR	international normalized ratio
KM	Kaplan–Meier
LASSO	least absolute shrinkage and selection operator
LCR	Lumphocyte to CRP ratio
LLET	low linear energy transfer
mbp	mean blood pressure
MDS	myelodysplastic syndrome
mGNRI	Modified Geriatric Nutrition Risk Index
mGPS	modified Glasgow Prognostic Score
MIMIC-IV	Medical Information Mart for Intensive Care
MSE	mean squared error
NF-κB	nuclear factor-kappaB
NGR	absneutrophil.glucose ratio
NLR	neutrophils to lymphocytes ratio
OASIS	Oxford Acute Severity of Illness Score
OS	overall survival
PAR	plateletcount.age ratio
PFS	progression-free survival
PGF	Plateletcount/glucose*followuptime
PLR	platelet-to-lymphocyte ratio
PNI	prognostic nutrition index
PT	prothrombin time
PTT	partial prothrombin time
ROC	receiver operator characteristic
RR	relative risk
SAPSII	Simplified Acute Physiology Score II
SD	standard deviation
SII	Systemic Immune-Inflammation Index
SIRI-RT	Serum Inflammation Risk Index for Radiation-Induced Secondary Malignancy
SIRS	Systemic Inflammatory Response Syndrome
SIS	systemic inflammation-immune status
SOFA	Sequential Organ Failure Assessment
SQL	Structured Query Language
TM	Thrombomodulin
VIF	variance inflation factor
WBC	white blood cells
